# scatterBrains: an open database of human head models and companion optode locations for realistic Monte Carlo photon simulations

**DOI:** 10.1117/1.JBO.28.10.100501

**Published:** 2023-10-05

**Authors:** Melissa M. Wu, Roarke Horstmeyer, Stefan A. Carp

**Affiliations:** a Duke University, Department of Biomedical Engineering, Durham, North Carolina, United States; b Massachusetts General Hospital, Harvard Medical School, Athinoula A. Martinos Center for Biomedical Imaging, Charlestown, Massachusetts, United States

**Keywords:** near-infrared spectroscopy, diffuse correlation spectroscopy, Monte Carlo, open database, head models

## Abstract

**Significance:**

Monte Carlo (MC) simulations are currently the gold standard in the near-infrared and diffuse correlation spectroscopy (NIRS/DCS) communities for generating light transport paths through tissue. However, realistic and diverse models that capture complex tissue layers are not easily available to all; moreover, manually placing optodes on such models can be tedious and time consuming. Such limitations may hinder the adoption of representative models for basic simulations and the use of these models for large-scale simulations, e.g., for training machine learning algorithms.

**Aim:**

We aim to provide the NIRS/DCS communities with an open-source, user-friendly database of morphologically and optically realistic head models, as well as a succinct software pipeline to prepare these models for mesh-based Monte Carlo simulations of light transport.

**Approach:**

Sixteen anatomical models were created from segmented T1-weighted magnetic resonance imaging (MRI) head scans and converted to tetrahedral mesh volumes. Approximately 800 companion scalp surface locations were distributed on each model, comprising full head coverage. A pipeline was created to place custom source and optical detectors at each location, and guidance is provided on how to use these parameters to set up MC simulations.

**Results:**

The models, head surface locations, and all associated code are freely available under the scatterBrains project on Github.

**Conclusions:**

The NIRS/DCS community benefits from having shared resources for conducting MC simulations on realistic head geometries. We hope this will make MRI-based head models and virtual optode placement easily accessible to all. Contributions to the database are welcome and encouraged.

## 1 Introduction

In the past several decades, Monte Carlo (MC) light simulations have emerged as the gold standard technique for simulating photon transport through tissue.^1^^,^^2^ Advances in these algorithms have allowed for modeling increasingly complex transport geometries or tissue types^3^^–^^9^ while drastically reducing the runtime through parallel or cloud computing architectures.^10^^–^^14^ Such developments have spawned new studies that use MC simulations to, among many other efforts, investigate potential tissue areas to image,^15^^–^^18^ determine the promise of new imaging modalities,^19^^–^^22^ optimize hardware in existing modalities,^23^^–^^28^ improve recovery of desired biomarkers,^29^^,^^30^ and further elucidate the physical principles behind light-tissue interaction.^31^^–^^35^ This list is far from exhaustive—the number of distinctive studies exploiting MC simulations has truly burgeoned across subfields in the past couple of decades.^2^ For details on methodology, the interested reader can peruse Zhu and Liu’s review paper^36^ on MC simulations in biological tissue, although much work has been developed since then as well.

Many works in the near-infrared and diffuse correlation spectroscopy (NIRS/DCS) communities have taken advantage of the Monte Carlo eXtreme (MCX)^37^ or mesh-based Monte Carlo (MMC)^38^ software packages written by Qianqian Fang and his team.^26^^,^^30^^,^^32^^,^^39^^–^^41^ Both packages have the capability to simulate arbitrary optode locations on any given complex, heterogeneous tissue model. However, many recent works using MCX/MMC have still used atlas-based head models (or even simpler slab or sphere geometries) while restricting the simulations to a fixed probe configuration at a single location.^24^^,^^30^^,^^32^^,^^42^^–^^44^ This is driven in part by difficulties in manually placing optodes and potentially limits the works’ investigative scope. Recently, both Nizam et al.^45^ and Bürmen et al.^46^ have acknowledged the need in the field for open reference datasets, as well as user-friendly multiple optode placement, for both machine learning training and validation of custom MC software. Both groups have provided datasets based on slab geometries with embedded absorbing targets of various shapes meant for training tomographic reconstruction algorithms. However, these are unrepresentative of realistic head, skull, and brain geometries and would not be as useful for the growing segment of the NIRS/DCS community targeting noninvasive detection of brain hemodynamics. Although a myriad of NIRS toolboxes exist,^47^ almost all are focused on signal or data analysis as their primary functionality and thus may often provide a single atlas-based head model. For users looking to run large-scale simulations considering many optode locations on diverse, realistic head models, such software may not be suitable.

In this work, we present scatterBrains, an open database of human head models along with companion optode locations of interest, and a toolbox to create specifications for running MC simulations of light transport, including the code to create input files compatible with MMC. The head models were derived from T1-weighted magnetic resonance imaging (MRI) scans acquired from healthy adult volunteers in the course of a previous study^30^ and subsequently segmented into five tissue layers. Each model was then “polished,” i.e., gaps or unwanted protrusions as a result of imperfect segmentation were manually smoothed over. The models were then converted into tetrahedral meshes using the iso2mesh toolbox.^48^ For the straightforward selection of a surface location of interest to run MMC simulations, 805 predefined scalp locations per model, comprising full head coverage, are provided for the user.^16^ Also included is a pipeline to automatically place user-specified optodes at any of these (or other) scalp locations and to write these and other parameters to MMC input files. Example post-processing for DCS is also given. The head models, their companion surface locations, and the processing pipeline comprise the scatterBrains open database.

## 2 Database Description and Methods

As mentioned above, the MRI structural scans that formed the basis for the head models in the toolbox were acquired in a previous research study approved by the Mass General Brigham Institutional Review Board (MGB IRB). Further, the MGB IRB approved the de-identification procedures and the sharing of the de-identified models in this open dataset.

The repository consists of 16 separate folders, 1 for each subject, as well as some functions and example scripts. Each subject folder contains files for the head model (both mesh- and volume-based) and the companion surface locations. Dependencies are described in Sec. 3.

### 2.1 Subject Head Models

The methodology for head model generation is described in our previous paper.^16^ Each model is segmented into five tissue layers representing the scalp, skull, cerebrospinal fluid, gray matter, and white matter. The head models were de-identified by removing the face area. The ears were also removed for more accurate optode placement across the head (users can contact the authors for models with the ears if needed). The details of the subject information is given in Table 1. Additional subject information regarding extracerebral thickness and cerebral sensitivity statistics are provided in Supplementary Material.

**Table 1:** Subject demographics.

Subject ID	Sex	Age	Race	Hispanic/Latino
1	M	24	More than one	Unknown/not reported
2	M	29	White	No
3	F	24	White	Yes
4	F	24	Asian	No
5	F	25	White	No
6	F	21	Black or African	No
7	F	39	White	No
8	F	32	White	No
9	F	23	Black or African	No
10	F	24	White	No
11	M	30	Asian	No
12	M	48	White	No
13	F	23	More than one	Yes
14	F	24	White	No
15	F	38	White	No
16	F	25	White	No

### 2.2 Companion Surface Locations

**Fig. 1:**
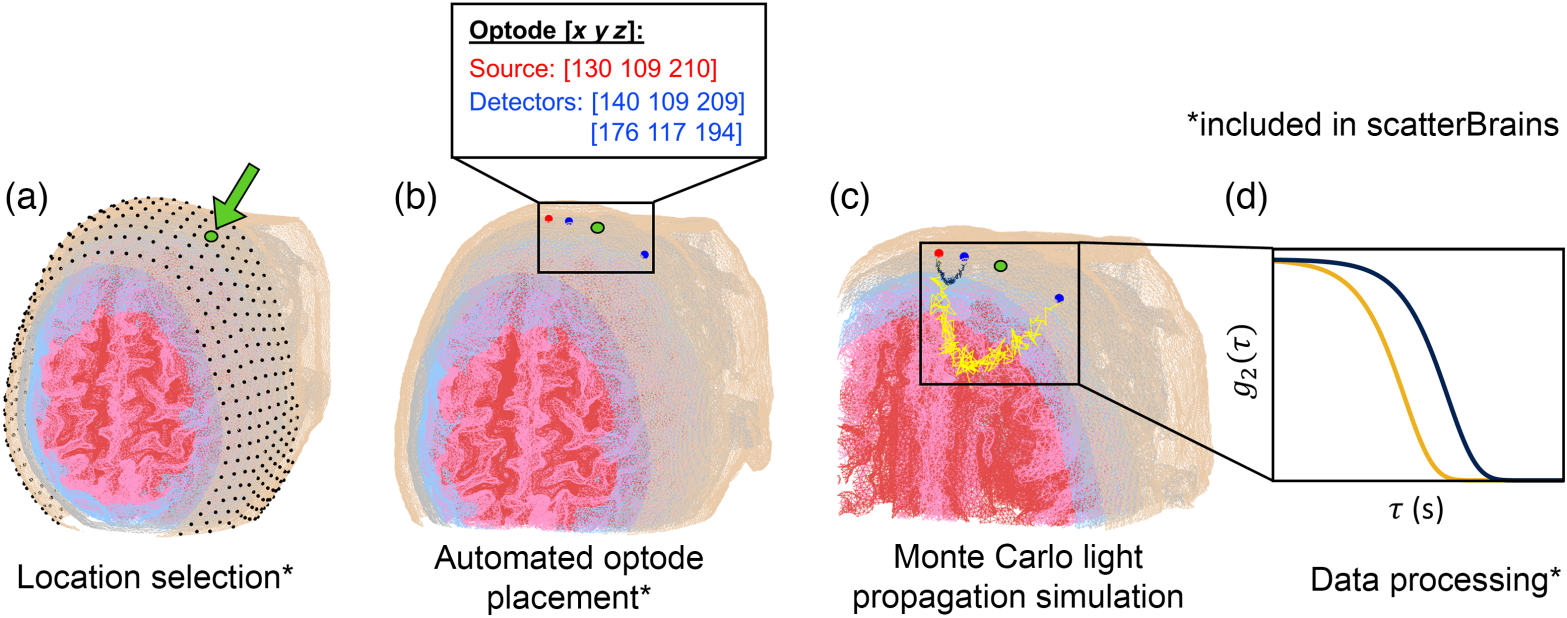
Pipeline demonstrating usage of scatterBrains functionality. (a) An example subject head with surface locations and a cross-section of the internal anatomy is shown. The subject head is lying supine with the de-identified face directed upward. The tan, gray, blue, pink, and red surfaces are the scalp, skull, cerebrospinal fluid, gray matter, and white matter, respectively. The user can select any one of the predefined surface locations or choose their own. (b) The optode placement algorithm will automatically generate source and detector positions at the surface location based on user-inputted distances. (c) A MC light propagation simulation can be run with the MMC software. scatterBrains provides all of the input information necessary for the simulation, and can also generate the input file directly passed to MMC. (d) The user can process the output photon trajectory data from the simulation. Analysis steps for DCS are also provided in the toolbox.

The methodology for surface location placement is also described in our previous work cited above. For this dataset, an additional 6 points near each ear were removed, resulting in a total of 805 surface locations for each model. A visualization of a subject head model and its companion surface locations is shown in Fig. 1(a).

### 2.3 Associated Functionality

**Fig. 2:**
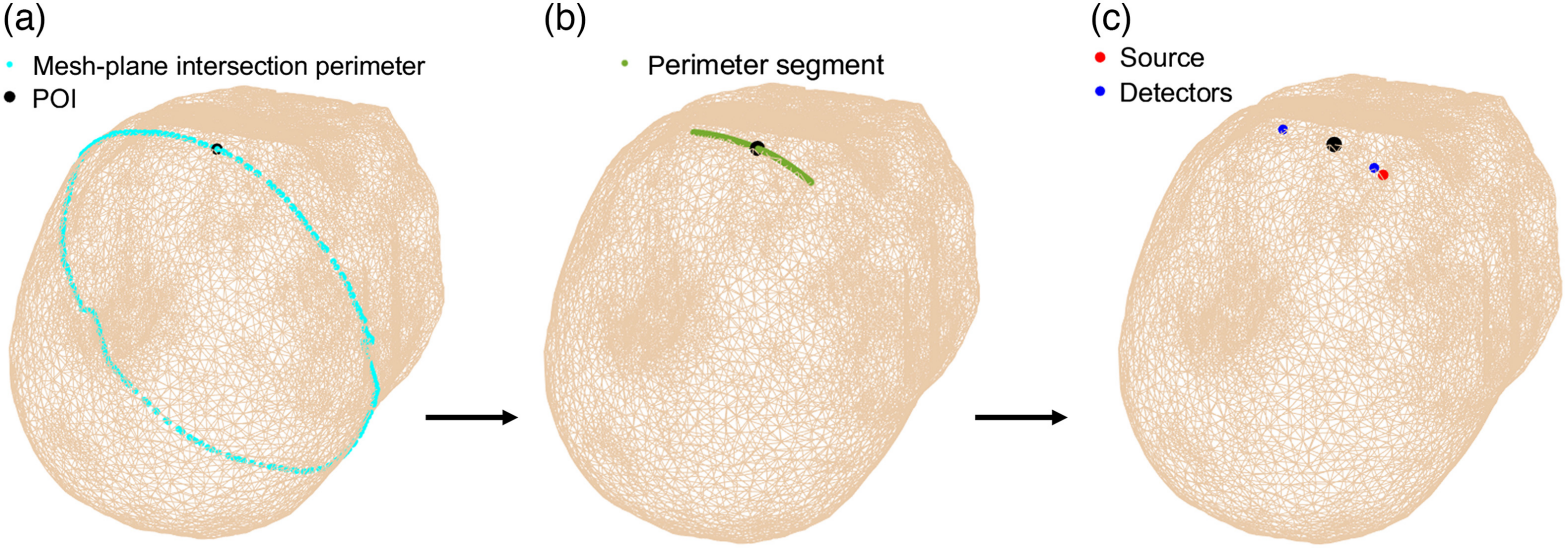
Optode placement pipeline for an example POI is shown. Here, the subject head is lying supine, with the facial features removed. The chosen POI is located above the subject eyebrow, slightly toward the subject’s right side. (a) A mesh-plane intersection is made with the plane defined by the POI and two points near the ears (not shown, but provided for the user). Its perimeter is extracted and shown. (b) An outer segment of this intersection perimeter surrounding the POI is taken. (c) Optodes are placed along this segment according to the desired distances inputted by the user, with the POI centered between the source and maximum detector distance. The red dot is the source, and the blue dots are detectors at 5 and 50 mm (the detector distance has been exaggerated for improved visualization).

Included in the toolbox is a pipeline for automatically placing sources and detectors at a given pre-defined head surface location [Fig. 1(b)]. We briefly describe the methodology here and denote the selected head surface location as the “point-of-interest” (POI). First, the intersection of a plane, defined by the POI and a point near each ear (also provided for the user), and the head mesh is taken. A segment of the intersection perimeter surrounding the POI is extracted, interpolated, and smoothed. The sources and detectors are then defined as points along this segment, per user-specified distances, with the POI centered between the source and furthest detector. Of note, the source–detector distances are calculated along the curved arc of the segment (not by the Euclidean distance)—we believe that this is more physically accurate for optical probes that are designed on a flat strip first and then wrapped around the head. Figure 2 shows the different steps of the optode placement pipeline. Users need only to input their desired POI (which can be visualized and selected from 1 of the provided 805 surface points) and the desired source–detector distances. The placement in the example below takes less than a second to run.

scatterBrains provides the code to write the optode locations, as well as other simulation parameters (e.g., optical properties for each tissue layer), directly to an input file, which can be passed to MMC along with the provided subject model. After running the simulation [Fig. 1(c)], the user can process the photon trajectory results as they wish [Fig. 1(d)]. The details of these steps and other remaining functionality are straightforward and presented in Sec. 3.

## 3 Usage

The repository hosting scatterBrains is publicly available on Github at https://github.com/wumelissa/scatterBrains and can be run out-of-the-box. We have tested the code on the Windows and Linux platforms—the repository and dependencies are cross-platform and should readily run on MacOS as well. The data and functions are written in Matlab and require the iso2mesh toolbox^48^ to be installed to run. The MMC package^38^ is needed to actually execute the MC simulation and provide photon history files for post-processing. The algorithm shown in Fig. 2 can be run on any of the surface locations in Fig. 1(a), for any combination of source–detector distances.

An end-to-end example script is provided for the user to demonstrate loading a model, selecting a POI, and placing optodes. The main steps of the complete pipeline demonstrated in the script are as follows:

Step 1. Select subject model
Step 2. Visualize model, and select surface location [Fig. 1(a)]
Step 3. Specify desired detector distances, and place optodes [automated, Fig. 1(b)]
Step 4. Specify additional MC parameters
Step 5. Specify desired tissue optical properties
Step 6. Run MMC [Fig. 1(c)]
Step 7. (DCS processing): Generate photon autocorrelations from MMC output [Fig. 1(d)]
Step 8. (DCS processing): Fit autocorrelations to semi-infinite model [Fig. 1(d)].

Steps 1–3 are described in Sec. 2. For Step 4, the code to generate the MMC input file (.json format) is provided, i.e., users only need to fill in their desired parameters in the Matlab script. The same applies for Step 5, for which the code is provided to generate an MMC optical properties file (.dat format). During Step 6, users can then run a simulation with their own MMC installation—MMC and iso2mesh are not redistributed in the scatterBrains repository because they are freely available. In Steps 7 and 8, post-processing code for generating diffuse correlation spectroscopy simulated measurements is also provided.

Aside from this end-to-end demo, additional example scripts are included to calculate the distance of the POI to the nearest brain tissue and to generate volume and surface meshes from the original segmented volume file. A Google groups forum is provided for community discussions and author support, although MMC-specific questions will be directed toward the MMC community support forum.

## 4 Discussion

In this work, we present an open dataset of realistic head models, companion surface locations, and code for user-friendly input file generation suitable for running MC simulations using the MMC package. As mentioned above, open datasets of slab tissue models with embedded blood vessels have recently been released for ease of large-scale MC simulations.^45^^,^^46^ However, these are primarily targeted toward diffuse optical tomography applications or smaller-scale spectroscopy measurements. For NIRS/DCS brain imaging, in which vessel reconstruction is not the main goal, modeling other tissue features (e.g., extracerebral layers, gyri and sulci of the brain) would be more valuable. Moreover, as measurements with larger source–detector separations (and thus a probed tissue area) are increasingly attained,^49^ larger and more comprehensive models are needed to capture photon paths.^32^ As mentioned previously, the NIRS community has many open-source toolboxes,^47^ primarily for signal and data analysis (as opposed to being database repositories); toolboxes such as AtlasViewer,^50^ NIRSTORM,^51^ and NIRS-SPM^52^ will generally provide a single model atlas, e.g., Colin27.^53^ If custom probe placement functionality is available in such toolboxes, it is generally restricted to graphical user interface (GUI)-level interaction, which may make preparing and running batch simulations tedious. We anticipate that our open dataset, surface locations, and optode placement functionality can be used in conjunction with these existing toolboxes for analyzing diverse subject models and surface locations. For DCS, toolboxes or datasets such as these have yet to be released—recent works involving MC simulations have often used layered slab or spherical models.^26^^,^^30^^,^^32^^,^^44^ Thus, for future DCS studies involving MC simulations, we expect that scatterBrains will be particularly useful. For both NIRS and DCS, we expect that our database will also be of use for large-scale machine learning training, in which thousands of optode configurations across the entire head can be automatically generated on a diverse set of subjects. Finally, we hope that scatterBrains can also be of use for cross-validating results from custom MC simulation software.

There are several means by which scatterBrains can be expanded upon in the near future. The current models are derived from predominantly female subjects in their 20s and 30s (driven by the demographics of those who responded to the study advertisement). A more diverse database in age, sex, and race/ethnicity would be beneficial for studying light transport in a wider range of subjects. Structural MRI scans are freely available to the public,^54^ so we anticipate no barrier in achieving such an improvement. With potential future expansion of the database, we additionally envision compiling “averaged” head models based on sex, age, etc., for use as references. On the pre-processing side, our group plans to eventually include both the image segmentation^55^ and surface location placement code (Secs. 2.1 and 2.2) available as part of the toolbox, so users can import their own MRI scans and subsequently use them as model inputs to MMC. For optode placement, we also aim to incorporate more complex optode placement schemes in the automated pipeline, as recent work has exploited non-linear setups in source–detector placement.^56^ With regard to DCS post-processing, we also plan to include MC fitting methods^30^ for DCS data, as well as other potential fitting schemes that have demonstrated improvement over the semi-infinite diffuse correlation equation. ^41^^,^^57^ Finally, because NIRS/DCS have also been used for blood flow monitoring in other tissue areas,^49^^,^^58^^,^^59^ in the long-term, we hope that scatterBrains will be a launching pad for open database generation of various organ models to use for MC simulations.

As an open database, we encourage users to contribute models and/or processing code to scatterBrains. Our goal is for this database to be easily accessible, user-friendly, and representative in its models. Although there is still work to be done in the future, we hope that collaboration with the NIRS/DCS community will help us toward realizing these aims.

## 5 Conclusion

We have presented an open database of human head models and companion surface locations derived from 16 adult subjects. For each model, the code for automatic optode placement at any of the 805 pre-defined surface locations can allow for straightforward and fast input file generation to MMC simulation software. We hope to expand this database and associated functionalities in the future.

## Code, Data, and Materials Availability

All code and data in this paper are publicly available at https://github.com/wumelissa/scatterBrains
