# Supplementary material for: scatterBrains: an open database of human head models and companion optode locations for realistic Monte Carlo photon simulations
Source: J Biomed Opt. 2023 Oct 5;28(10):100501. doi: 10.1117/1.JBO.28.10.100501 (PMC10557038; doi:10.1117/1.JBO.28.10.100501)
Supplement: Supplementary file 1 [file JBO_028_100501_SD001.docx]

**Supplementary Content: *scatterBrains*, an open database of human head models and companion optode locations for realistic Monte Carlo photon simulations**

Melissa M. Wu, Roarke Horstmeyer, Stefan A. Carp

The tables below provide supplemental information in different areas of the head for each subject. The user should bear in mind that these values are averaged over their respective areas, and that tissue thickness at any one particular location will vary.

Table S1 provides the total extracerebral thickness (E.C.T.), as well as the cerebral sensitivity (Sens.) at a 30 mm source-detector separation. The latter is defined as the percent recovered cerebral blood flow increase from a 50% ground truth increase. Details of the cerebral sensitivity calculation can be found in our previous work^16^.

**Table S1** Average (total) extracerebral thickness (E.C.T.) and 30 mm cerebral sensitivity (Sens.) in different areas of the head. E.C.T units are in mm, and sensitivity units are in percentage.

| **Subject ID** | **Frontal** | | **Occipital** | | **Temporal** | | **Parietal** | |  |
| --- | --- | --- | --- | --- | --- | --- | --- | --- | --- |
|  | **E.C.T. (mm)** | **Sens. (%)** | **E.C.T. (mm)** | **Sens. (%)** | **E.C.T. (mm)** | **Sens. (%)** | **E.C.T. (mm)** | **Sens. (%)** | |
| **1** | 18.96 | 5.14 | 18.63 | 5.32 | 16.59 | 8.05 | 20.06 | 3.90 | |
| **2** | 15.06 | 13.23 | 15.33 | 12.38 | 15.84 | 9.61 | 18.09 | 5.80 | |
| **3** | 14.33 | 16.66 | 13.63 | 20.77 | 13.15 | 22.85 | 16.23 | 13.81 | |
| **4** | 13.53 | 20.79 | 12.94 | 22.13 | 13.79 | 18.31 | 16.50 | 12.48 | |
| **5** | 12.76 | 22.44 | 14.70 | 15.86 | 13.43 | 19.30 | 15.10 | 15.09 | |
| **6** | 14.04 | 18.30 | 17.23 | 7.31 | 15.15 | 12.13 | 19.59 | 4.10 | |
| **7** | 14.67 | 15.44 | 14.29 | 16.65 | 13.82 | 18.13 | 16.66 | 12.87 | |
| **8** | 18.26 | 6.58 | 14.42 | 14.93 | 15.36 | 11.23 | 19.09 | 5.83 | |
| **9** | 14.80 | 14.18 | 18.44 | 5.93 | 16.69 | 7.84 | 19.93 | 4.82 | |
| **10** | 13.25 | 20.13 | 14.59 | 15.70 | 13.76 | 16.05 | 16.88 | 10.65 | |
| **11** | 14.75 | 15.98 | 15.41 | 11.84 | 14.89 | 12.56 | 18.42 | 6.40 | |
| **12** | 15.81 | 11.34 | 17.15 | 7.10 | 18.26 | 4.77 | 19.16 | 4.76 | |
| **13** | 14.49 | 16.59 | 15.11 | 13.51 | 13.40 | 18.91 | 17.65 | 8.44 | |
| **14** | 12.89 | 22.28 | 11.17 | 29.54 | 11.17 | 29.70 | 15.06 | 18.48 | |
| **15** | 14.35 | 17.17 | 13.48 | 21.76 | 12.80 | 22.96 | 16.57 | 14.28 | |
| **16** | 15.18 | 13.30 | 14.98 | 13.90 | 13.81 | 17.44 | 17.24 | 9.93 | |

Tables S2 and S3 provide the scalp and skull thicknesses in different areas of the head, respectively.

**Table S2** Average scalp thickness in different areas of the head. All units are in mm.

| **Subject ID** | **Frontal** | **Occipital** | **Temporal** | **Parietal** |
| --- | --- | --- | --- | --- |
| **1** | 6.22 | 9.37 | 9.72 | 8.81 |
| **2** | 6.87 | 8.13 | 10.37 | 8.87 |
| **3** | 5.41 | 6.76 | 6.84 | 7.13 |
| **4** | 4.94 | 7.13 | 7.84 | 8.11 |
| **5** | 5.87 | 7.65 | 8.11 | 8.25 |
| **6** | 6.10 | 9.64 | 9.58 | 10.37 |
| **7** | 5.71 | 7.58 | 7.63 | 7.39 |
| **8** | 5.58 | 7.43 | 7.64 | 7.31 |
| **9** | 6.45 | 9.50 | 10.43 | 9.47 |
| **10** | 5.65 | 7.78 | 7.86 | 8.46 |
| **11** | 6.09 | 8.54 | 9.86 | 11.13 |
| **12** | 7.27 | 8.86 | 11.31 | 8.93 |
| **13** | 5.45 | 7.33 | 7.84 | 7.90 |
| **14** | 4.96 | 5.37 | 6.19 | 6.57 |
| **15** | 5.01 | 5.37 | 6.12 | 6.07 |
| **16** | 5.68 | 7.61 | 8.19 | 8.15 |

**Table S3** Average skull thickness in different areas of the head. All units are in mm.

| **Subject ID** | **Frontal** | **Occipital** | **Temporal** | **Parietal** |
| --- | --- | --- | --- | --- |
| **1** | 10.82 | 7.45 | 4.92 | 9.31 |
| **2** | 6.38 | 5.24 | 3.39 | 7.39 |
| **3** | 6.70 | 5.02 | 4.04 | 6.30 |
| **4** | 6.34 | 4.07 | 4.01 | 5.89 |
| **5** | 4.90 | 5.25 | 3.56 | 4.85 |
| **6** | 5.82 | 5.85 | 3.88 | 7.27 |
| **7** | 6.71 | 4.97 | 4.18 | 6.34 |
| **8** | 9.76 | 5.24 | 5.70 | 9.16 |
| **9** | 6.40 | 7.05 | 4.48 | 8.28 |
| **10** | 5.62 | 5.02 | 4.12 | 5.84 |
| **11** | 6.52 | 5.17 | 3.21 | 5.09 |
| **12** | 6.44 | 6.54 | 5.05 | 8.04 |
| **13** | 6.93 | 6.10 | 3.79 | 7.61 |
| **14** | 5.90 | 4.13 | 3.06 | 6.01 |
| **15** | 7.05 | 6.36 | 4.64 | 7.71 |
| **16** | 7.53 | 5.55 | 3.73 | 6.64 |
